# The Importance of Different Forms of Social Capital for Happiness in Europe: A Multilevel Structural Equation Model (GSEM)

**DOI:** 10.1007/s11482-022-10097-1

**Published:** 2022-08-22

**Authors:** Lucía Gómez-Balcácer, Noelia Somarriba Arechavala, Patricia Gómez-Costilla

**Affiliations:** 1 Department of Applied Economics, University of Valladolid, María Zambrano Campus, Plaza de la Universidad 1, 40005 Segovia, Spain; 2 Department of Economic Analysis, University of Valladolid, María Zambrano Campus, Plaza de la Universidad 1, 40005 Segovia, Spain

**Keywords:** Social capital, Happiness, Trust, Social interaction, Europe, Multilevel structural equations model

## Abstract

This paper investigates the effects of different dimensions of social capital ​on happiness of Europeans. Unlike other studies, a categorical principal component analysis (CATPCA) is applied to obtain the dimensions of social capital. The data used come from the ninth wave of the European Social Survey (ESS), year 2018. Happiness is modelled using a multilevel structural equations model (GSEM) by country to study the role of social capital in Europeans' happiness, when controlling for the effect of factors such as gender, unemployment, age, low income, higher education, and living with a partner. Social capital is measured as a multidimensional concept composed of institutional trust, social trust, social ties and voluntary association, civility and sense of belonging, and religiosity. Among the results, we found that the five dimensions that build social capital have a positive impact on happiness. In addition to the positive effects of social capital, the control variables have the expected impact. In a context marked by growing individualism and social isolation, the results of this work can guide policy makers in using the dimensions of social capital to increase the subjective well-being of the population.

## Introduction

The study of happiness or subjective well-being is by no means a recent research topic. It is an expanding field with a large and growing number of papers (Bruni & Porta, 2016; Graham & Behrman, 2009; Stutzer & Frey, 2010; Veenhoven, 2009, among others). Most of these papers have focused on the role played by factors such as income, education, and health among others, in explaining happiness. Other issues, such as social and institutional variables, have received less attention in the literature, even though they have shown promise as significant determinants in a world marked by COVID-19, and in which governments have been forced to adopt containment strategies to curb the spread of the virus. In particular, social distancing and isolation have posed a major challenge to human interaction. Social connections, interactions and relationships are an essential part of human evolution, and their absence has led to states of anxiety, depression and mental disorders, among other problems, that have reduced individuals’ well-being. In the current context, social capital becomes an essential tool to reverse the negative effects of the crisis, through cooperation, solidarity, trust and reciprocity. In fact some studies, such as, Becchetti et al., 2008; Bjørnskov, 2003; Helliwell & Putnam, 2004; Klein, 2013; Winkelmann, 2009, find that social capital has a positive effect on happiness. In contrast, Ram (2010) finds a tenuous link between social capital, as represented by generalized trust, and happiness. In our paper, we explore the role of social capital in determining happiness in the European context. Working with a broad sample of European countries, our analysis is enriched by the diversity of cultures, values and traditions, which is a plurality that in turn influences the configuration of social capital.

One variable that lies very close to the concepts of social capital and happiness is marital status. Previous studies show that marital status also proves key when exploring happiness, and that it is a source of social support for many people (Hudson, 2006; Veenhoven, 2005). However, the impact of marriage on happiness and social capital is not always positive. Indeed certain works, such as Luhmann et al. (2012), show that married people became no happier than when they were single. Kislev (2020) demonstrated that social capital increases happiness among single people more than among married people and also that single people obtain greater happiness than married people with equal social capital. In our work, we look at the effect of cohabitation on happiness, considering that modern societies are characterized by the decline of marriage and the rise of informal cohabitation. Stutzer and Frey (2006) argue that marriage provides more happiness or satisfaction benefits than cohabitation, whereas Soons and Kalmijn (2009) find that in countries where cohabitation is institutionalized, the happiness gap between married and cohabiting people disappears or even reverses.

At the empirical level, in the literature dealing with the determinants of subjective well-being, income appears as one of the key variables when explaining happiness. It should be noted that in the lowest income deciles the effect of income on happiness is accentuated (Deaton, 2008; Graham & Behrman, 2009). Frey and Stutzer (2002) find that in the richest and most developed countries, income is positively correlated with happiness, although the increase in happiness decreases over time. In the same vein, Clark et al. (2008) suggest that subjective well-being depends not only on the absolute level of income, but also on the comparison of this level with that of other people, especially those who share similar socioeconomic characteristics. However, Mentzakis and Moro (2009) find that relative income has little effect in the lower income group, but that among the higher income group there is a large and significant effect. As regards socioeconomic status, the level of employment or unemployment also appears as an important variable in explaining happiness. In general, unemployment is negatively related to the level of happiness. According to Di Tella et al. (2001), when controlling for other factors, the unemployed are less happy than the employed in the United States and several European countries. Studer and Winkelmann (2014) highlights that an increase in the unemployment rate is associated with a sharp decline in subjective well-being in Germany. However, Clark and Oswald (1994) find that the unemployed in Great Britain are happier in districts where the unemployment rate is higher, which could be explained by a reduction in the stigma associated with being unemployed. Ahn et al. (2004) find that the effect of unemployment is greater in countries where the labour market functions worse and where there is a lower level of protection for the unemployed.

As for education, there is no consensus vis-à-vis the effects of education on subjective well-being. For example, Hayo and Seifert (2003) find that education has a positive impact on happiness in eastern European transition countries. Using data from the United States and the United Kingdom in the period from the early 1970s to the late 1990s, Blanchflower and Oswald (2004) find a positive relationship between education and happiness. In contrast, Inglehart and Klingemann (2000) work with a large sample of countries and find no significant effect of education proxies on life satisfaction. From a sample of British workers, Clark (1996) reports that people with more education are unhappier at work, with one possible explanation being that highly educated people have higher job expectations that are more difficult to meet.

Another determinant highlighted in the happiness literature refers to health, which in most studies is measured by subjective health; that is, measured as self-reported evaluations of general or physical health (Langeland, 2014). Along the same lines, Subramanian et al. (2005) find a strong relationship between well-being and subjective health in the USA. Using data from the United States, Latin America and Russia, Carol Graham (2008) finds that people who report having a good state of health are happier. Pierewan and Tampubolon (2015) study 47 countries in Europe and find that happiness and health are positively correlated, such that the authors construct a multivariate multilevel model with happiness and health as dependent variables. According to the study by Pierewan and Tampubolon (2015, p. 238), “happier people tend to be healthier, even after controlling for individual, regional and national covariates”.

This article also analyses the effect of different sociodemographic variables, such as age and gender, on subjective well-being. Most studies suggest the existence of a U-shaped relationship between age and happiness (Blanchflower & Oswald, 2008; Clark, 2003). People tend to be happier when they are younger and as they get older than when they are middle-aged. However, other empirical studies have yielded contradictory results. In his analysis of data from the 2006 Gallup World Poll, Deaton (2008), the winner of the 2015 Nobel Prize in Economics, concluded that the results of the relationship between age and life satisfaction differ across countries. For its part, the effect of gender is less clear in the literature. For example, Zweig (2015) argues that there is no significant difference between women's and men's happiness in a sample of 73 countries. Stevenson and Wolfers (2009) use data from the United States and Western Europe show that women report better levels of well-being in more developed countries, where opportunities for women are greater, which has an impact on their levels of subjective well-being. In contrast, Mencarini and Sironi (2012) conclude that women who are involved in a higher percentage of domestic activities have lower levels of subjective well-being, regardless of whether or not they live in a developed country.

In order to explore the determinants of happiness in Europe, and focusing particular attention on social capital, this paper uses data from the latest wave (year 2018)—at the time of writing this article—of the European Social Survey (ESS) to analyze the determinants of happiness in 24 European Union countries.

As will be seen later, the relationship between the two concepts—social capital and happiness—is by no means new. However, this work makes two contributions to the literature. First, it quantifies social capital using categorical principal component analysis (CATPCA), a multivariate analysis technique that allows us to explore the concept of social capital and its components. Secondly, the social capital model derived from the previous analysis enables us to incorporate, in addition to the traditional determinants used in different works (gender, unemployment, age, income, education, etc.), a further five dimensions of social capital obtained from CATPCA (institutional and social trust, social ties and voluntary association, religion, sense of belonging, and civic mindedness), which are introduced in order to analyze the results in a large sample of European countries. Furthermore, given that the analysis is carried out in a spatial context, a multilevel fixed effects model is proposed for the different countries involved in the analysis, using a multilevel structural equation model, where social capital is defined as a latent class model based on the components derived from the CATPCA.

Our work pursues a twofold objective: first, to identify different dimensions of social capital and, second, to ascertain which dimensions of social capital have a greater significant impact on happiness—if indeed they have any effect on it at all. With these objectives in mind, the rest of the paper is organized as follows. The second section provides a brief review of the literature on the relationship between social capital and happiness, with special emphasis on the dimensions of social capital identified in the study. The third section presents the data, the methodology and the different components derived from the categorical principal components analysis (CATPCA). In the following section, we model happiness for our set of countries using the multilevel structural equations model (GSEM) technique by countries in order to study the role played by social capital in happiness when controlling for the effect of factors such as gender, unemployment, age, low income, higher education and living with a partner. Finally, the article closes with a discussion of the main results and conclusions arising from this study.

## A Multidimensional Approach to Measuring Social Capital

Social capital is a controversial concept, mainly due to its ambiguity and the difficulties involved in measuring it (Häuberer, 2010). The definitions of social capital by Bourdieu, Coleman and Putnam have contributed greatly to disseminating the concept (Rogošić & Baranović, 2016). Bourdieu (1986, p. 248) defines social capital as “the resources available to an individual as a function of his or her membership in a group”. Coleman (1988, p. 302) understands social capital as “a variety of aspects of social structures, information channels, obligations and expectations, sets of rules and sanction systems that facilitate or inhibit the actions of actors”. Although the roots of the concept go back to Bourdieu and Coleman, it was the 1993 work of Robert Putnam that brought social capital clearly into the public debate. Putnam (1993, p. 167) defined social capital as "those aspects of social organizations, such as networks, norms and trust, that enable action and cooperation for mutual benefit". The definitions of Bourdieu, Coleman and Putnam revolve around trust, networks and norms of reciprocity and reliability, with each author placing greater emphasis on one or the other variable (Hommerich & Tiefenbach, 2018).

The dimension “trust” can be thought of as a belief about the trustworthiness of other people; that is, about how others are likely to behave. The second dimension, “networks”, measures social relationships directly through intensity of contact, frequency of interactions and social network characteristics. Finally, the dimension “norms” uses measures of membership in specific volunteer organizations and is generally treated as an indirect measure of social ties or norms, which are believed to be fostered by volunteer organizations. Apart from these three components or dimensions, an in-depth review of the literature on the subject reveals the wide variety of aspects encompassed by the concept of social capital (Bjørnskov, 2006; Frey & Stutzer, 2002; Helliwell & Putnam, 2004; Klein, 2013, among others). The diversity of variables used to represent or assess social capital in empirical works responds to the multidimensional nature of the concept (Bjørnskov, 2008). In fact, we note that most studies do not consider all dimensions of social capital simultaneously, focusing on one or another dimension.

In the literature addressing the determinants of social capital, trust appears as one of the main determinants, with the results generally showing a positive relationship between trust and subjective well-being (Rodríguez-Pose & von Berlepsch, 2014). For example, Ekici and Koydemir (2014) analyze the changes Turkey underwent between 1999 and 2008 and find that trust has a significant influence on well-being. Using data from Luxembourg, Klein (2013) also finds a positive and significant relationship between different measures of subjective well-being (happiness and life satisfaction) and institutional trust. In Europe, there are works that link institutional trust and individual happiness, and that report a positive relationship between the two variables (Frey & Stutzer, 2002; Hudson, 2006; Tavits, 2008). In a similar vein, Bjørnskov (2003) uses data from European countries to analyze, among other aspects, the relationship between social capital, measured through generalized trust and a factor analysis-derived variable on generalized trust, civic participation and perceived corruption, and the level of life satisfaction. The results show that both social capital variables exert a positive and significant influence on happiness. In addition, Leung et al. (2011) finds that institutional trust continues to have an effect on well-being even when other dimensions of social capital are taken into account. In contrast, the work of Ram (2010) suggests that social trust has little effect on happiness.

In terms of social ties, interaction with peers, friends and family is felt to facilitate the individual’s social integration. Using data from the 2004 German Socio-Economic Panel, Winkelmann (2009) finds that social capital is an important predictor of well-being levels, as measured by life satisfaction. As proxy measures of social capital, he uses participation in different activities, ranging from attending cultural events to voluntary participation in social or political organizations. Rodríguez-Pose and von Berlepsch (2014) find that social connections and participation in social events are positively related to happiness. Powdthavee (2008) finds a positive relationship between social contacts and subjective well-being. However, Bjørnskov (2008) conducts a cross-sectional analysis for the United States and finds that this relationship is not significant. Pichler (2006) argues that the greater an individual's involvement in non-profit and non-political organizations, the greater the well-being reported by individuals. Helliwell and Putnam (2004) reach a similar conclusion for the United States; participation in clubs or organizations has a positive influence on happiness at the macro level, although they find no significant correlation at the micro level.

Closely linked to associationalism, participation in religious communities seems a priori to play an essential role in shaping individuals’ social capital and, therefore, their happiness. Religious social capital is usually proxied by the frequency of church attendance (Hayo, 2004; Helliwell & Putnam, 2004). Several authors, such as Hayo and Seifert (2003) and Helliwell and Putnam (2004), consider that church attendance facilitates the creation of social capital at the community level, thereby increasing attendee happiness. The results of these papers confirm Putnam's argument about the feedback effects between the different dimensions of social capital. The social capital created by frequent church attendance fosters trust between people, which leads to an increase in social capital and, consequently, in well-being. In this sense, (Ferrer, 2001) argues that belonging to a religious community strengthens social relations and facilitates the redistribution of resources, i.e., it fosters social cohesion and mutual aid. Stavrova et al. (2013) find that the effect of religion on subjective well-being is greater in highly religious cultures, whereas the relationship is negative in cultures that value atheism. In this sense, Lun and Bond (2013) argue that the impact of religion on well-being is affected by social hostility towards religion.

Place attachment is a multifaceted concept that incorporates different facets of the link between place and people and involves the relationship between knowledge and beliefs and behaviors and actions in reference to a place (Scannell & Gifford, 2017). However, in the literature we rarely find examples that relate happiness and sense of belonging as a form of social capital. Studying the sense of belonging in Europe can help to promote a shared European identity that facilitates a cohesive future among the members of the European Union (EU). Wellman et al. (2001) argue that the sense of belonging derived from social interactions between people is an important element in generating social capital and fostering individuals’ participation in the community.

The results to emerge from the literature review confirm the multidimensional nature of social capital. Approaching the measurement of social capital by means of multivariate analysis techniques, such as the CATPCA, mentioned above, can therefore shed light on the dimensions of this concept and how they relate to each other. In order to analyze whether social capital is a determining factor in happiness and in which particular aspects thereof it might be so, and given its multidimensional nature, we first perform a CATPCA analysis to identify the dimensions of social capital. We then estimate an econometric model to gauge what impact these different components of social capital, as well as control variables such as age or gender, might have on individual well-being by means of multilevel structural equation modelling.

## An Empirical Analysis of the Impact of Social Capital on Happiness

In this section “Data”, an initial brief description of the survey used—the European Social Survey (ESS)—is presented. The following section “Application of Categorical Principal Components Analysis (CATPCA) to the Study of Social Capital in Europe” describes the variables used in the CATPCA analysis, which is made up of 20 indicators related to different aspects of social capital and which shows the main results obtained in the analysis. Finally, in section “Multilevel Generalized Structural Equation Modelling in the Study of Happiness”, an econometric model is proposed to study the question of whether social capital is a key source when determining happiness. For this purpose, a multilevel structural equation model is estimated, and social capital is introduced into this model as a latent variable.

### Data

The data used are taken from the ninth wave of the European Social Survey (ESS), year 2018. The core module of the survey is used to collect changes in a wide range of social variables, including public and social trust, interest and participation in politics, socio-political orientations, government and its effectiveness, social, political and moral values, social exclusion, national, ethnic and religious loyalty, well-being, health, safety, demographic and socio-economic factors, among others (Portela et al., 2013). It provides data at country and regional level, which is very useful when applying multilevel models, and its main objective is to lay the foundations for generating social and attitudinal indicators that are not only recognized in academic circles but, as with other indicators in the field of economics, are widely accepted in the political and social sphere and can be used in comparative analysis and decision making. This survey has been selected as a starting point because it provides a vast amount of data for a large sample of countries. We work with most EU28 countries, except Greece, Luxembourg and Malta, who did not participate in the 2018 survey, and Romania, whose data are not yet integrated.

Considering the multidimensional nature of social capital, we applied categorical principal component analysis to reduce an initial set of 20 variables, in line with the reviewed literature, to a smaller number of dimensions of social capital. This analysis will allow us to explore in greater depth the main dimensions of social capital and its configuration in a spatial area such as the European Union.

### Application of Categorical Principal Components Analysis (CATPCA) to the Study of Social Capital in Europe

The purpose of applying CATPCA analysis is to reduce the original set of variables to a smaller set of uncorrelated components without losing the information found in the original variables (Kemalbay & Korkmazoğlu, 2014). The main differences between this technique and classical principal component analysis (PCA) concern the type of variables used and the relationship between them (Linting et al., 2007). PCA requires variables to be measured on a metric scale, while CATPCA uses any type of variable (Linting & van der Kooij, 2012). In addition, it allows nonlinear relationships between the studied variables to be operated and revealed (Molina & De los Monteros Pérez, 2010). In our study, we used ordinal and nominal variables, and since we do not know whether there is a linear relationship between them it is necessary to use CATPCA. Other studies have also used this method to transform nominal and ordinal variables into smaller data sets, thus helping to interpret the results (Acik-Toprak, 2009; Comim & Amaral, 2013; Saukani & Ismail, 2019).

This paper has identified 20 potential variables to study social capital in view of the previously consulted literature (see Table 1), with most of the variables being ordinal, except for the last two, which are nominal. Table 1 shows the definition of the variables as well as the principal statistics thereof (mean, standard deviation, number of observations).

**Table 1:** Social capital variables

Variable	Description	Mean/%	Std Dev	n
Emotional attachment to the country (atchctr)	Scale 0 to 10: Not emotionally attached at all (0) and very emotionally attached (10)	7.86	2.182	42,260
Emotional attachment to Europe (atcherp)	Scale 0 to 10: Not emotionally attached at all (0) and very emotionally attached (10)	5.96	2.567	41,565
People with whom you can talk about intimate and personal matters (inprdsc)	Scale 0 to 6: None (0), 1 (1), 2 (2), 3 (3), 4–6 (4), 7–9 (5), 10 or more (6)	2.72	1.466	41,922
Important to do what is said and follow the rules (ipfrule)	Scale 1 to 6: Not at all like me (1), Very much like me(6)	3.75	1.384	41,288
It is important to help people and to care about the welfare of others (iphlppl)	Scale 1 to 6: Not at all like me (1), Very much like me(6)	4.81	1.001	41,616
Most people try to be fair, or try to take advantage of you (pplfair)	Scale of 0 to 10: People mostly look out for themselves (0) and most people try to be fair (10)	5.65	2.274	42,141
Most of the time people helpful or mostly looking out for themselves (pplhlp)	Scale 0 to 10: Most people try to take advantage of me (0) and people mostly try to be helpful (10)	5.01	2.311	42,285
Most people can be trusted or you can't be too careful (ppltrst)	Scale 0 to 10: You can't be too careful (0) and most people can be trusted (10)	5.05	2.462	42,338
How often do you attend religious services, other than on special occasions? (rlgatnd)	Scale 1 to 7: Never (1), Every day (7)	2.54	1.497	42,120
How religious are you? (rlgdgr)	Scale 0 to 10: Not at all religious (0) and very religious (10)	4.50	3.140	42,021
Participate in social activities compared to other people of the same age (sclact)	Scale 1 to 5: Much less than most (1), and much more than most (5)	2.71	0.940	41,764
How often socially meet with friends, relatives or colleagues (sclmeet)	Scale 1 to 7: Never (1), and every day (7)	4.78	1.586	42,315
Trust in politicians (trstplt)	Scale of 0 to 10: No confidence (0) and total confidence (10)	3.58	2.438	41,736
Trust in political parties (trstprt)	Scale of 0 to 10: No confidence (0) and total confidence (10)	3.55	2.406	41,623
Trust in the country's parliament (trstprl)	Scale of 0 to 10: No confidence (0) and total confidence (10)	4.41	2.614	41,537
Trust in the legal system (trstlgl)	Scale of 0 to 10: No confidence (0) and total confidence (10)	5.291	2.715	41,630
Trust in the European Parliament (trstep)	Scale of 0 to 10: No confidence (0) and total confidence (10)	4.48	2.551	39,702
Trust in the police (trstplc)	Scale of 0 to 10: No confidence (0) and total confidence (10)	6,43	2.488	42,161
Have you worked in another organization or association in the last 12 months (wrkorg)	Dichotomous: No (0) or Yes (1)	0.15	0.357	42,279
Have you worked in a political party or action group in the last 12 months (wrkprty)	Dichotomous: No (0) or Yes (1)	0.04	0.186	42,298

Source: ESS (2018). Own elaboration

The CATPCA results support our retaining five dimensions, which explain 53% of the variance in the 20 items investigated, thus indicating reasonable adjustment. The proportion of variance explained by a component is its eigenvalue divided by 20, which is the number of variables in this study (Saukani & Ismail, 2019). In this analysis, we ignore dimensions greater than five because their contribution to the total variance accounted for is very small. Table 2 shows the percentage explained by each additional dimension and the eigenvalue. All of the latter are greater than one.

**Table 2:** Model summary rotation

Dimension	Variance accounted for	Variance accounted for
	Total (eigenvalue)	% of variance
1	3,726	18.630
2	2.255	11.275
3	2.047	10.235
4	1.700	8.500
5	1.678	8.390
Total	11.405	57.025
Alfa de Cronbach total: 0.960		
a. Rotation method: Varimax with Kaiser normalization		

Source: ESS (2018). Own elaboration

The alpha coefficient of the five components is high, suggesting that the items have relatively high internal consistency, with a reliability coefficient of 0.70 or higher being considered an acceptable measure in most situations (Tavakol & Dennick, 2011). Varimax rotation is an orthogonal rotation method in which the axes are kept perpendicular so that the resulting factors are not correlated with each other (De Campos et al., 2020). This facilitates the meaning of the interpretation of the selected components. This is the most commonly used rotation and is also taken into account when the objective is to reduce the number of uncorrelated variables to a smaller number.

Table 3 shows the connection between the dimensions of social capital proposed in this paper and the work of Bourdieu, Coleman and Putnam, who are key authors in social capital research.

**Table 3:** Relating authors and dimensions of social capital

Authors	Dimensions of social capital (institutional trust, social trust, social ties and associationalism, civility and sense of belonging, and religiosity)
Coleman (1988), Putnam (1993)	Institutional trust (1)
Coleman (1988), Putnam (1993)	Social trust (2)
Bourdieu (1986), Coleman (1988), Putnam (1993)	Social ties and associationalism (3)
Coleman (1988), Putnam (1993, 2000)	Civility and sense of belonging (4)
Coleman (2003), Putnam (2000)	Religiosity (5)

Source: Own elaboration

As can be seen, in their works most of these authors consider the aspects we have analyzed in this article and that are relevant when explaining social capital.

With regard to the first two dimensions of the analysis –institutional and social trust– Coleman (1988) stresses that trust is key to the creation of social capital, and Putnam (1993) highlights the importance of social values (especially trust) in fostering the proper functioning of the economic system and in a high level of political integration.

In relation to social ties and voluntary association, Bourdieu (1986) emphasizes the existence of networks of relationships that provide each of their members with the support of collectively owned capital. Coleman (1988) stresses that information channels (meeting with peers, friends or family), facilitate the development and welfare of society through coordination and cooperation among the members of a group. Along the same lines, Putnam (1993) also emphasizes the importance of social networks (especially voluntary associations).

As for civility and sense of belonging, Coleman (1988) stresses that social capital implies effective norms and sanctions (that society has strong norms and clear sanctions). In a similar vein, Putnam's (1993) concept of social capital also points to the importance of moral obligations and norms.

Together with the literature on trust, networks, civility and sense of belonging, there is growing literature linking social capital to religion. Putnam (2000) argues that religious communities are important sources of social capital. Similarly, Smidt and Smidt (2003) says that membership and participation in religious organizations increases social capital through relationship building. Other authors have also analyzed the relationship between different dimensions of social capital and religion (see, for example, Berggren & Bjørnskov, 2011; Halman & Luijkx, 2006; Kaasa, 2013). Does religion encourage cooperation and positive attitudes towards others? Berggren and Bjørnskov (2011) find that religiosity is negatively related to trust, especially because religious people tend to distrust those who do not share their beliefs. In contrast, Halman and Luijkx (2006) find that higher levels of religiosity increase the level of trust in institutions, which in turn leads to an increase in well-being. Similarly, Kaasa (2013) also finds a positive relationship between religion and trust.

Table 4 presents the classification of the variables according to the rotated component loading in the dimensions of social capital in this study:

**Table 4:** Rotated component loadings

Dimension					
Variables	1	2	3	4	5
Trust in politicians	0.859	0.078	0.074	-0.039	0.058
Trust in political parties	0.853	0.067	0.087	-0.040	0.057
Trust in the country's parliament	0.817	0.142	0.096	0.041	0.014
Trust in the legal system	0.741	0.233	0.062	0.101	-0.059
Trust in the European Parliament	0.703	0.027	0.057	0.103	-0.002
Trust in the police	0.611	0.258	0.013	0.214	-0.014
Most people try to take advantage of you or try to be fair	0.167	0.858	0.124	0.030	-0.024
Most of the time, people are helpful or mostly look out for themselves	0.170	0.812	0.074	0.030	0.002
Most people can be trusted or you can't be too careful	0.223	0.802	0.124	0.016	-0.020
How often you meet socially with friends, relatives or colleagues	-0.012	0.070	0.785	0.074	-0.103
Taking part in social activities compared to others of the same age	0.020	0.033	0.765	0.032	-0.072
How many people with whom you can discuss intimate and personal matters	0.082	0.181	0.549	0.011	-0.079
Having worked in another organization or association over the last 12 months	0.114	0.086	0.499	-0.034	0.055
Having worked in a political party or action group over the last 12 months	0.054	-0.040	0.348	-0.047	0.102
How emotionally attached to [country]	0.076	0.055	-0.088	0.810	0.079
How emotionally attached to Europe	0.188	0.044	0.034	0.731	-0.102
Important to do what you are told and to follow rules	0.024	-0.058	-0.066	0.449	0.165
Important to help people and to care for others’ well-being	-0.044	0.086	0.305	0.424	0.101
How often you attend religious services apart from special occasions	-0.001	-0.032	0.035	0.048	0.905
How religious you are	0.042	0.001	-0.034	0.207	0.870

Source: ESS (2018). Own elaboration

Results point to the existence of five components. These dimensions are related with institutional trust, social trust, social ties and associationalism, civility and sense of belonging, and religiosity, as can be seen in the matrix of rotated factor loadings in Table 4:

1. The first dimension, institutional trust, which is particularly relevant in the definition of social capital, is made up of variables that expressly refer to citizens' trust in public institutions, such as trust in politicians, political parties, the country's parliament, the judicial system, the European Parliament, the police and the judiciary.
2. The second dimension focuses on social trust, which is basically composed of the degree of trust existing between individuals in a society, proxied by indicators relating to whether people are fair, helpful or trustworthy.
3. The third component is related to the dimension of social ties and voluntary association. It is related to participation in social activities, associations and/or political parties, the frequency of meetings with friends, family or colleagues and whether they have people they trust to discuss personal and intimate issues.
4. The following component is connected with civic-mindedness and the sense of belonging, captures emotional attachment to the country and to Europe, as well as concern for the welfare of other citizens.
5. Finally, the religiosity dimension is measured by attendance at religious services and the degree of religiosity of the respondent.

The diversity of cultures, ethnicities, institutions and traditions that characterize the European continent leads us to think that there are differences in the social capital of the four main European macro-regions (North, South, East and West). As far as the trust components are concerned, we find important regional differences. We observe that social and institutional trust is higher in the northern countries. When evaluating the third dimension of social capital, we observe that social ties and voluntary association is lower in the east. The dimension related to civic-mindedness and sense of belonging is relatively homogeneous across all macro-regions in Europe. Compared to Northern, Western and Southern Europe, the dimension of religiosity presents higher values in the east.

These dimensions are included in our econometric model by means of a latent variable as discussed below. As regards the scores of the social capital dimensions, it should be noted that they are standardized values and that all of them are expected to have a positive impact on both happiness and social capital.

### Multilevel Generalized Structural Equation Modelling in the Study of Happiness

We used structural equations modelling (SEM) to test the effect of social capital dimensions and control variables on happiness. A two-level (multilevel) model with individuals nested within the country is estimated. SEM models are statistical multivariate models and are accompanied by the graphical representation of causal relationships, the formulation of hypotheses and the concatenation of effects between variables. These are models that allow the relationship between latent and observed variables to be studied. An observed variable is one that can be measured directly, such as age or gender, whereas a latent variable can only be measured indirectly through a set of observed variables (Bartholomew et al., 2008). The direction of causality in the relationships between the latent variable and the indicators or observed variables may differ (Fayers & Hand, 2002; Van Beuningen & Schmeets, 2013). The indicators may form the construct, with the direction of causality being formative or causal; in other words, from the observed variables to the construct. In the applied model, the construct or latent variable measuring social capital is considered to be the result of the five dimensions obtained from the CATPCA analysis. Below, we present the dependent variable and the control variables that we use in the model. The population is weighted with the pweight variable. This weighting corrects for the fact that most countries participating in the EES have different population sizes but similar sample sizes.

#### Dependent Variable

Our study variable is happiness, with the study of happiness having become one of the fastest growing fields of research in economics over the last few decades (Clark et al., 2008; Kahneman & Krueger, 2006). The subjective well-being approach is based on directly asking people about their well-being, and the dominant approach to analyzing subjective well-being data is through ordinal parametric methods (ordered probit or logit) or linear regression (OLS) (MacKerron, 2012). In our model, happiness is modelled by an ordered logit, one of the most commonly used models when working with happiness (Alesina et al., 2004; Blanchflower & Oswald, 2004; Ferrer-i-Carbonell, 2005). Specifically, the survey used asks the following question: "Taking everything into account, how happy would you say you are?". Respondents were asked to rate their happiness on a scale of 0 to 10, with (0) being extremely unhappy and (10) extremely happy. The average of the variable happiness was 7.4, and the standard deviation 1.92 (*n* = 42,295).

#### Explanatory Variables

In addition to individuals’ scores in the dimensions of social capital in the form of a latent model, we introduce a series of control variables that are commonly applied in this type of work, as referred to in the introductory section. The following Table 5 shows their description and the expected impact on happiness in view of the studies consulted.

**Table 5:** Control variables

Variable	Description	Mean/%	Std Dev	n	Expected direction on Happiness
Gender	Dummy variable that takes the value 1 when the respondent is female	0.54	0.50	42,467	+
Unemployment	Dummy variable that takes the value 1 if the individual has ever been unemployed and looking for a job for a period longer than three months	0.28	0.45	42,204	_
Age	Individuals´year of age	51.29	18.68	42,297	_
Low income	Dummy variable that takes the value 1 when the respondent belongs to the first income decile	0.10	0.31	34,077	_
Education	Years of full-time education completed	13.04	4.16	41,864	+
Living with a partner	Dummy variable that takes the value 1 when the respondent lives with his or her partner	0.81	0.39	42,340	+
Health	Dummy variable that takes the value 1 when the respondent self-rated his or her health as very good or good	0.65	0.48	42,418	+

Source: Own elaboration

**Fig. 1:**
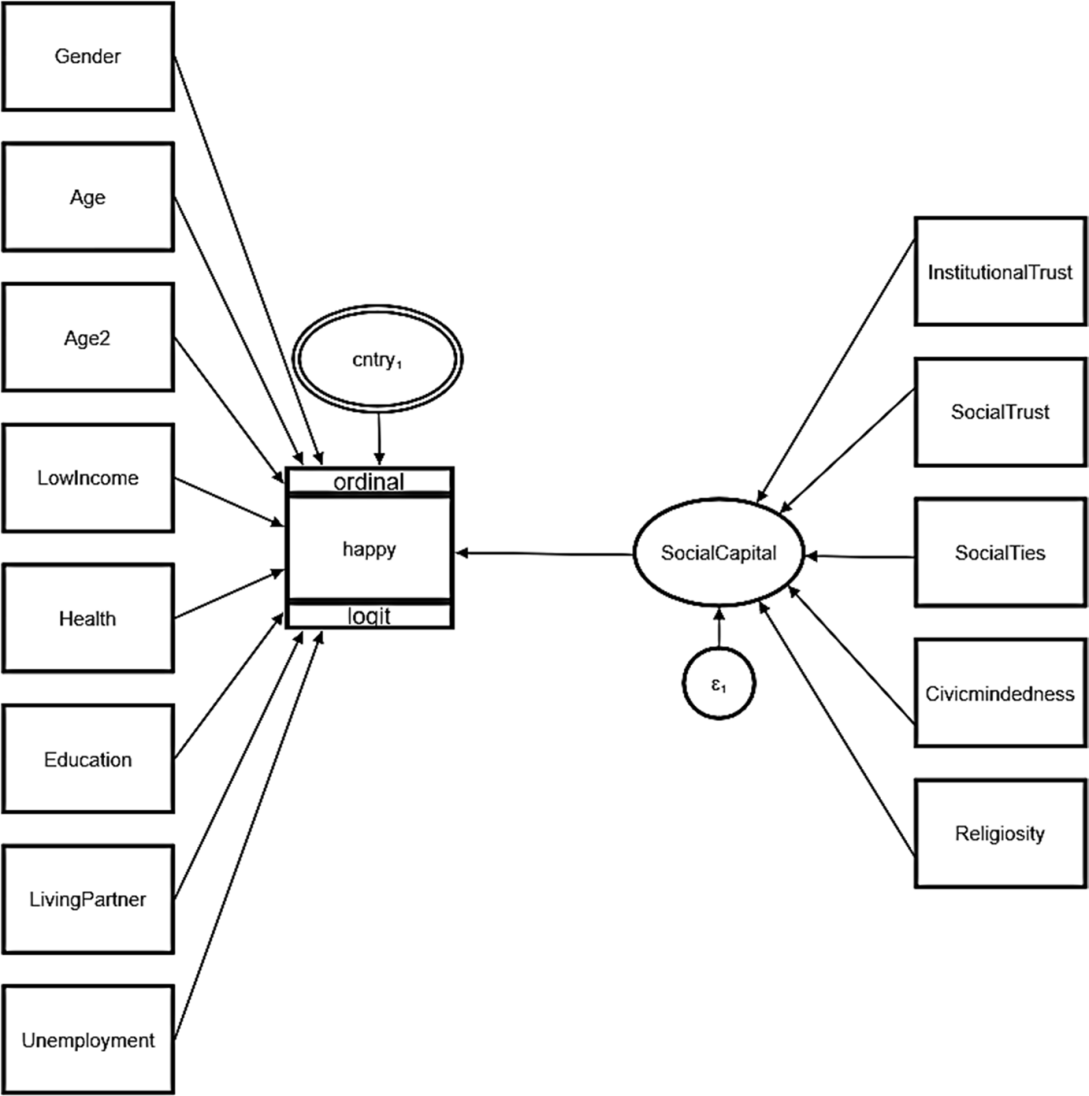
Model GSEM. Source: Own elaboration with the Stata17 program

With this set of variables, together with the derived dimensions of social capital, we propose the following modelling (Fig. 1) in which we can see that age is also squared, in order to study the quadratic U-shape effect (Clark & Oswald, 1994).

Taking advantage of the data structure, we apply a multilevel model, specifically a two-level model, with level 1 corresponding to individuals and level 2 to countries. Furthermore, given the nature of our happiness variable expressed on a scale where 0 is extremely unhappy and 10 extremely happy, an ordered multilevel logit model is proposed. The ordinal logistic regression model is usually used when the dependent variable is ordinal. In other words, it is used to facilitate the interaction of dependent variables (which have multiple ordered levels) with one or more independent variables. Social capital adopts the format of a latent variable in the diagram. These variables can be thought of as a composite score of other variables and they are represented by ovals. By proceeding in this way, we can study the impact of each of the proposed dimensions on our configuration of social capital.

## Results

The GSEM is a key tool for causal mediation analysis, although it does make strong assumptions and offers few tests to assess goodness of fit. The Akaike information criterion (AIC) and Bayesian information criterion (BIC) are used to measure the performance of the model proposed in this study. Table 6 shows the results of the empty model, the model with control variables, and the full model, reporting odds ratios, AIC and BIC. Control variables and social capital variables are significant in both models. When comparing the AIC and BIC values of the empty model and the full model, the results indicate that the full model is better able to capture the relationship between happiness and social capital.

**Table 6:** GSEM (Multilevel regression analyses)

Model (Dependent variable: How happy are you?) (Odds ratios)			
Independent variable	Model 1 (*n* = 40,411)	Model 2 (*n* = 31,258)	Model 3 (*n* = 31,258)
Gender		1.081***	1.043**
Age		0.972***	0.980***
Age squared		1.000***	1.000***
Education		1.014***	0.989***
Health		2.600***	2.254***
Unemployment		0.733***	0.779***
Low income		0.555***	0.604***
Living with a partner		1.382***	1.391***
Institutional trust			1.341***
Social trust			1.427***
Social ties and voluntary association			1.472***
Civic-mindedness and sense of belonging			1.525***
Religiosity			1.055***
AIC	151,745.9	113,635.1	109,744.8
BIC	151,840.6	113,793.7	109,945.2

*P*-values: *p < 0.01; **p < 0.05; ***p < 0.001

Source: Own elaboration

Seven control variables were included in the model, since previous research has found these to be associated with happiness: age, gender, unemployment, education, cohabitation, low income household income, and self-rated health. In addition, the five dimensions that symbolize social capital in our work were added. These were institutional trust, social trust, social ties and voluntary association, civic-mindedness and sense of belonging, and religiosity.

The results for the block of control variables indicated a significant effect, supporting previous works that found these variables to be important in predicting happiness. When gender-related information was taken into account, a significant gender gap emerged, with women being more likely to have a higher level of happiness than men. Overall, age is negatively associated with happiness, although quadratic age has a positive association, confirming the existence of a U-shaped relationship between age and happiness (Pierewan & Tampubolon, 2015). In contrast, living together as a couple was associated with greater happiness, a result that is in line with the paper by Musick and Bumpass (2012), which identifies living together as a more beneficial factor for happiness and self-esteem in the USA.

Socioeconomic characteristics were introduced into the estimation and many of them were found to be a source of happiness inequalities. For example, 10% of the households with the lowest income were more likely to report being unhappy. This result shows that income matters for happiness when households have low incomes. Being unemployed was significantly associated with a lower probability of being happy. In addition, a higher level of education was found to be related to a lower levels of happiness. The results support the work of Veenhoven (2005), who identifies that education does not generally make people happier. Along these lines, Clark and Oswald (1996) argue that higher education can make people more ambitious, which could reduce life satisfaction, as higher aspirations are more difficult to fulfill.

Among the control variables, the variable that has the greatest impact on happiness is health. Respondents who reported better health were more likely to be happy. The results for health are consistent with the findings of Gerdtham and Johannesson (2001), whose work reported a significant and positive relationship between happiness and good health. Similarly, Subramanian et al. (2005) worked with U.S. data and found that individuals who are healthier tend to be happier.

All dimensions of social capital are significant in our analysis. As in previous research (e.g. Helliwell & Wang, 2010), the positive and significant coefficient indicates that an increase in institutional trust, one of the key elements of social capital, makes individuals happier. Our model revealed a positive impact of social trust on happiness. Social ties also play an important role in facilitating social change and connecting groups (Kao, 2004). Consistent with most findings in the literature on social ties and associationalism, the results show that a strong social network of friends and participation in associations and political groups makes individuals happier. As regards civic-mindedness and sense of belonging, we found a positive relationship between happiness and this dimension of social capital. In contrast to other works, such as Bjørnskov (2008), in our analysis we found that civility has a significant positive impact on happiness. Helliwell and Barrington-Leigh (2012) also incorporate variables of sense of belonging to a community, province or country and find that these variables are highly significant and positively related to subjective well-being. Religiosity, proxied by frequency of church attendance and degree of belief, is associated with greater happiness. Barro and McCleary (2003) find a significant and positive association between religious freedom and happiness. In this sense, Frey and Stutzer (2002) conclude that believing in God positively affects happiness, which is supported by our model.

## Discussion and Conclusion

Happiness is one of the driving forces of human life and is a concept that arouses significant scientific and social interest (Ram, 2010). One of the main objectives of our work was to evaluate the role of social capital in explaining happiness. Following the work of different authors, we provide a broader definition of social capital, dividing the concept into five dimensions. As the findings of our study suggest, trust, participation in associations, civility and religion are positively associated with subjective well-being. Using the models developed, we identify significant relationships between happiness and the five dimensions. We have found that social ties lead to higher levels of happiness. Human beings establish various types of interpersonal relationships throughout their lives so that they need social support to be happy. Authors such as, for example, Rodriguez-Llanes et al. (2013) find that social support is one of the most important resilience factors after stressful events. By feeling listened to, understood, supported and respected, our well-being increases. Therefore, social support can certainly be a useful tool to fight against the effects of COVID in society. However, we should not forget that the relationship between social ties and happiness may vary according to gender, age and socioeconomic position, as these determinants are associated with different responsibilities and resources. The results of the study are evidence of these variations. In relation to social support, religion also has a positive effect on happiness. What could be the explanation? The main factor seems to be the strong social support that churches offer to their members. It seems that it is not so much the fact of believing in something, but the fact of belonging and participating in a community that supports and comforts its members. Religion, with its rituals, promotes the sharing of experiences and the feeling of belonging to a community. The results are particularly relevant in a context marked by COVID-19, in which trust in institutions in many countries has been worryingly eroded. This study provides valuable empirical research on social capital by examining the effects of various forms of social capital on well-being that are significantly influenced by the sociocultural context of Europe. In recent years there has been a great deal of public and political debate about immigration, ethnic diversity and social cohesion (Cheong et al., 2007). Many people argue that the rise of populism in Europe and elsewhere is due to people feeling marginalized, distrusted and generally unhappy. In the current climate, trust in institutions and a sense of belonging are of vital importance to ensure peaceful coexistence between the different EU states. The EU is a multicultural space and, therefore, in order to guarantee a happy coexistence, group identities must be protected, without forgetting to respect individual rights. European decision-makers must contribute to the creation of a European identity so as to ensure the EU survives. By strengthening trust in institutions and fostering a self-identity, the EU could avoid a situation similar to that experienced with Brexit. The UK was for a long time one of the most Eurosceptic countries in the EU, which resulted in a weak European identity. Carl et al. (2019) find that the British people’s weak sense of European identity was a key factor in the Brexit vote. In recent years, the Czech Republic or certain eastern European countries such as Hungary have also shown a strong distrust of EU institutions. All of this highlights the need to strengthen ties between EU members. Understanding the links between social capital and subjective happiness at the individual and aggregate levels allows us to further the already extensive research on happiness.

Our research does, however, evidence certain limitations. First, our intention was to cover all EU countries. Nevertheless, the data series was not available for all countries. Second, the evidence of the results is limited to the set of countries studied, although it would be interesting to establish a division by regions at the European level so as to determine whether social capital interacts with happiness differently depending on the area analyzed. In addition, prominent among the control variables is the effect of health on happiness, such that we believe it would be interesting to develop a bivariate multilevel model to study the effect of social capital on both variables. Moreover, the results show a significant gender gap, such that in future work it would be interesting to explore whether this gap might be explained by social capital.

## References

[CR1] Acik-Toprak N (2009). Civic engagement in Europe: A multilevel study of the effect of individual and national determinants on political participation.

[CR2] Ahn, N., García, J. R., & Jimeno, J. F. (2004). The impact of unemployment on individual well-being in the EU. European Network of Economic Policy Research Institutes, Working Paper, 29.

[CR3] Alesina A, Di Tella R, MacCulloch R (2004). Inequality and happiness: Are Europeans and Americans different?. Journal of Public Economics.

[CR4] Barro RJ, McCleary RM (2003). Religion and economic growth across countries. American Sociological Review.

[CR5] Bartholomew DJ, Steele F, Moustaki I (2008). Analysis of multivariate social science data.

[CR6] Becchetti L, Pelloni A, Rossetti F (2008). Relational goods, sociability, and happiness. Kyklos.

[CR7] Berggren N, Bjørnskov C (2011). Is the importance of religion in daily life related to social trust? Cross-country and cross-state comparisons. Journal of Economic Behavior & Organization.

[CR8] Bjørnskov, C. (2003). The happy few: Cross-country evidence on social capital and life satisfaction. *Kyklos,**56*(1), 3–16. 10.1111/1467-6435.00207

[CR9] Bjørnskov, C. (2006). The multiple facets of social capital. *European Journal of Political Economy, 22*(1), 22–40. 10.1016/j.ejpoleco.2005.05.006

[CR10] Bjørnskov C (2008). Social trust and fractionalization: A possible reinterpretation. European Sociological Review.

[CR11] Blanchflower DG, Oswald AJ (2004). Well-being over time in Britain and the USA. Journal of Public Economics.

[CR12] Blanchflower DG, Oswald AJ (2008). Is well-being U-shaped over the life cycle?. Social Science & Medicine.

[CR13] Bourdieu P (1986). The forms of capital. Cultural Theory: An Anthology.

[CR14] Bruni, L., & Porta, P. L. (2016). Happiness and quality of life reconciled. *Handbook of Research Methods and Applications in Happiness and Quality of Life* (pp. 1–19). 10.4337/9781783471171.00005

[CR15] Carl N, Dennison J, Evans G (2019). European but not European enough: An explanation for Brexit. European Union Politics.

[CR16] Cheong P, Edwards R, Goulbourne H, Solomos J (2007). Immigration, Social Cohesion and Social Capital: A Critical Review. Critical Social Policy - CRIT SOC POLICY.

[CR17] Clark AE (1996). Job satisfaction in Britain. British Journal of Industrial Relations.

[CR18] Clark AE (2003). Unemployment as a social norm: Psychological evidence from panel data. Journal of Labor Economics.

[CR19] Clark AE, Oswald AJ (1994). Unhappiness and unemployment. The Economic Journal.

[CR20] Clark AE, Oswald AJ (1996). Satisfaction and comparison income. Journal of Public Economics.

[CR21] Clark AE, Frijters P, Shields MA (2008). Relative income, happiness, and utility: An explanation for the Easterlin Paradox and other puzzles. Journal of Economic Literature.

[CR22] Coleman JS (1988). Social capital in the creation of human capital. American Journal of Sociology.

[CR23] Comim F, Amaral PV (2013). The human values index: Conceptual foundations and evidence from Brazil. Cambridge Journal of Economics.

[CR24] De Campos CI, Pitombo CS, Delhomme P, Quintanilha JA (2020). Comparative analysis of data reduction techniques for questionnaire validation using self-reported driver behaviors. Journal of safety research.

[CR25] Deaton A (2008). Income, health, and well-being around the world: Evidence from the Gallup World Poll. Journal of Economic Perspectives.

[CR26] Di Tella R, MacCulloch RJ, Oswald AJ (2001). Preferences over inflation and unemployment: Evidence from surveys of happiness. American Economic Review.

[CR27] Ekici T, Koydemir S (2014). Social capital, government and democracy satisfaction, and happiness in Turkey: A comparison of surveys in 1999 and 2008. Social Indicators Research.

[CR28] Fayers PM, Hand DJ (2002). Causal variables, indicator variables and measurement scales: An example from quality of life. Journal of the Royal Statistical Society: Series A (statistics in Society).

[CR29] Ferrer JN (2001). Revisioning transpersonal theory: A participatory vision of human spirituality.

[CR30] Ferrer-i-Carbonell A (2005). Income and well-being: An empirical analysis of the comparison income effect. Journal of Public Economics.

[CR31] Frey BS, Stutzer A (2002). What can economists learn from happiness research?. Journal of Economic Literature.

[CR32] Gerdtham U-G, Johannesson M (2001). The relationship between happiness, health, and socio-economic factors: Results based on Swedish microdata. The Journal of Socio-Economics.

[CR33] Graham C (2008). Happiness and health: Lessons and questions for public policy. Health Affairs.

[CR34] Graham, C, & Behrman, J. (2009). *How Latin Americans assess their quality of life: Insights and puzzles from novel metrics of well-being*. 1–21.

[CR35] Halman L, Luijkx R (2006). Social capital in contemporary Europe: Evidence from the European Social Survey. Portuguese Journal of Social Science.

[CR36] Häuberer, J. (2010). *Social Capital Theory*.

[CR37] Hayo B, Seifert W (2003). Subjective economic well-being in Eastern Europe. Journal of Economic Psychology.

[CR38] Hayo, B. (2004). *Happiness in Eastern Europe*.

[CR39] Helliwell, J. F., & Barrington-Leigh, C. P. (2012). How much is social capital worth?. In The social cure (pp. 55–71). Psychology Press.

[CR40] Helliwell JF, Putnam RD (2004). The social context of well-being. Philosophical Transactions of the Royal Society of London. Series B, Biological Sciences.

[CR41] Helliwell J, Wang S (2010). Trust and wellbeing. International Journal of Wellbeing.

[CR42] Hommerich C, Tiefenbach T (2018). Analyzing the Relationship Between Social Capital and Subjective Well-Being: The Mediating Role of Social Affiliation. Journal of Happiness Studies.

[CR43] Hudson J (2006). Institutional trust and subjective well-being across the EU. Kyklos.

[CR44] Inglehart, R., & Klingemann, H.-D. (2000). Genes, culture, democracy, and happiness. En *Culture and subjective well-being.* (pp. 165–183). The MIT Press. 10.1007/978-90-481-2352-0_2

[CR45] Kaasa A (2013). Religion and social capital: Evidence from European countries. International Review of Sociology.

[CR46] Kahneman D, Krueger AB (2006). Developments in the measurement of subjective well-being. Journal of Economic Perspectives.

[CR47] Kao G (2004). Social capital and its relevance to minority and immigrant populations. Sociology of Education.

[CR48] Kislev E (2020). Social capital, happiness, and the unmarried: A multilevel analysis of 32 European countries. Applied Research in Quality of Life.

[CR49] Kemalbay G, Korkmazoğlu ÖB (2014). Categorical principal component logistic regression: a case study for housing loan approval. Procedia-Social and Behavioral Sciences.

[CR50] Klein C (2013). Social capital or social cohesion: What matters for subjective well-being?. Social Indicators Research.

[CR51] Langeland, E. (2014). Sense of Coherence BT - Encyclopedia of Quality of Life and Well-Being. In A. C. Michalos (Ed.), *Research* (pp. 5831–5833). Dordrecht: Springer.

[CR52] Leung A, Kier C, Fung T, Fung L, Sproule R (2011). Searching for happiness: The importance of social capital. Journal of Happiness Studies.

[CR53] Linting M, van der Kooij A (2012). Nonlinear principal components analysis with CATPCA: A tutorial. Journal of Personality Assessment.

[CR54] Linting M, Meulman JJ, Groenen PJF, van der Koojj AJ (2007). Nonlinear principal components analysis: Introduction and application. Psychological Methods.

[CR55] Luhmann, M., Hofmann, W., Eid, M., & Lucas, R. E. (2012). Subjective well-being and adaptation to life events: A meta-analysis. En *Journal of Personality and Social Psychology* (Vol. 102, Número 3, pp. 592–615). American Psychological Association. 10.1037/a0025948 10.1037/a0025948 PMC3289759 22059843

[CR56] Lun VM-C, Bond MH (2013). Examining the relation of religion and spirituality to subjective well-being across national cultures. Psychology of Religion and Spirituality.

[CR57] MacKerron G (2012). Happiness economics from 35 000 feet. Journal of Economic Surveys.

[CR58] Mencarini L, Sironi M (2012). Happiness, housework and gender inequality in Europe. European Sociological Review.

[CR59] Mentzakis E, Moro M (2009). The poor, the rich and the happy: Exploring the link between income and subjective well-being. The Journal of Socio-Economics.

[CR60] Molina ÓM, De los Monteros Pérez E (2010). Rotación en análisis de componentes principales categórico: un caso práctico. Metodología de encuestas.

[CR61] Musick K, Bumpass L (2012). Reexamining the case for marriage: Union formation and changes in well-being. Journal of Marriage and Family.

[CR62] Pichler F (2006). Subjective quality of life of young Europeans. Feeling happy but who knows why?. Social Indicators Research.

[CR63] Pierewan AC, Tampubolon G (2015). Happiness and health in Europe: A multivariate multilevel model. Applied Research in Quality of Life.

[CR64] Portela M, Neira I, del Salinas-Jiménez M, M. (2013). Social Capital and Subjective Wellbeing in Europe: A New Approach on Social Capital. Social Indicators Research.

[CR65] Powdthavee N (2008). Putting a price tag on friends, relatives, and neighbours: Using surveys of life satisfaction to value social relationships. The Journal of Socio-Economics.

[CR66] Putnam RD (2000). Bowling alone: The collapse and revival of American community.

[CR67] Putnam, R. (1993). The prosperous community: Social capital and public life. *The american prospect*, *13*(Spring), Vol. 4. Available online: http://www.prospect.org/print/vol/13 (accessed 7 April 2003).

[CR68] Ram R (2010). Social capital and happiness: Additional cross-country evidence. Journal of Happiness Studies.

[CR69] Rodríguez-Pose A, von Berlepsch V (2014). Social capital and individual happiness in Europe. Journal of Happiness Studies.

[CR70] Rodriguez-Llanes JM, Vos F, Guha-Sapir D (2013). Measuring psychological resilience to disasters: are evidence-based indicators an achievable goal?. Environmental Health.

[CR71] Rogošić, S., & Baranović, B. (2016). Social capital and educational achievements: Coleman vs. Bourdieu. *Center for Educational Policy Studies Journal, 6*(2), 81–100.

[CR72] Saukani N, Ismail NA (2019). Identifying the Components of Social Capital by Categorical Principal Component Analysis (CATPCA). Social Indicators Research.

[CR73] Scannell L, Gifford R (2017). The experienced psychological benefits of place attachment. Journal of Environmental Psychology.

[CR74] Smidt, C. E., & Smidt, S. F. C. E. (Eds.). (2003). *Religion as social capital: Producing the common goo*d. Baylor University Press. (pp.33-47)

[CR75] Soons JPM, Kalmijn M (2009). Is marriage more than cohabitation? Well-being differences in 30 European countries. Journal of Marriage and Family.

[CR76] Stavrova O, Fetchenhauer D, Schlösser T (2013). Why are religious people happy? The effect of the social norm of religiosity across countries. Social Science Research.

[CR77] Stevenson B, Wolfers J (2009). The paradox of declining female happiness. American Economic Journal: Economic Policy.

[CR78] Studer R, Winkelmann R (2014). Reported happiness, fast and slow. Social Indicators Research.

[CR79] Stutzer A, Frey BS (2006). Does marriage make people happy, or do happy people get married?. The Journal of Socio-Economics.

[CR80] Stutzer A, Frey BS (2010). Recent advances in the economics of individual subjective well-being. Social Research.

[CR81] Subramanian SV, Kim D, Kawachi I (2005). Covariation in the socioeconomic determinants of self rated health and happiness: A multivariate multilevel analysis of individuals and communities in the USA. Journal of Epidemiology and Community Health.

[CR82] Tavakol M, Dennick R (2011). Making sense of Cronbach’s alpha. International Journal of Medical Education.

[CR83] Tavits M (2008). Representation, corruption, and subjective well-being. Comparative Political Studies.

[CR84] Van Beuningen J, Schmeets H (2013). Developing a social capital index for the Netherlands. Social Indicators Research.

[CR85] Veenhoven R (2005). Inequality of happiness in nations. Journal of Happiness Studies.

[CR86] Veenhoven, R. (2009). How do we assess how happy we are? Tenets, implications and tenability of three theories. *Happiness, Economics and Politics: Towards a Multi-Disciplinary Approach*, *August*, 45–69. 10.4337/9781849801973.00009

[CR87] Wellman B, Haase AQ, Witte J, Hampton K (2001). Does the internet increase, decrease, or supplement social capital?: Social networks, participation, and community commitment. American Behavioral Scientist.

[CR88] Winkelmann R (2009). Unemployment, social capital, and subjective well-being. Journal of Happiness Studies.

[CR89] Zweig JS (2015). Are women happier than men? Evidence from the Gallup World Poll. Journal of Happiness Studies.

